# Efficacy of Genicular Nerve Block in Patients with Osteoarthritis: A Comparative Study with and without Fluoroscopy Assistance

**DOI:** 10.1055/s-0044-1793825

**Published:** 2024-12-21

**Authors:** Nour Aliman El Majzoub Said, Alexandre Barbieri Mestriner, Heloisa Sungaila, Enzo Salviato Mameri, Carlos Eduardo Silveira Franciozi, Marcelo Seiji Kubota

**Affiliations:** 1 Grupo do Joelho e Artroscopia, Departamento de Ortopedia e Traumatologia, Escola Paulista de Medicina, Universidade Federal de São Paulo (Unifesp), São Paulo, SP, Brasil

**Keywords:** fluoroscopy, nerve block, osteoarthritis, knee, pain, visual analog pain scale

## Abstract

**Objective** To compare the efficacy of fluoroscopy as an auxiliary method in genicular nerve block (GNB) with block guidance by anatomical parameters, without imaging aid, in reducing pain.

**Methods** A total of 23 patients underwent fluoroscopy-guided or anatomical parameter-based GNBs. We applied the Western Ontario and McMaster Universities' Osteoarthritis Index (WOMAC) and the Visual Analog Scale (VAS) for pain at 6 time points (preblock, and after 1 hour, 24 hours, 7 days, 28 days, and 90 days).

**Results** The mean age of the sample was of 64.5 ± 4.8 years, and the mean Body Mass Index (BMI), of 31.4 ± 6.1 Kg/m ^2^ ; 16 subjects (69.6%) were women. The WOMAC pain subscale showed a significant reduction ( *p* < 0.05) in pain in both groups at all time points. This reduction was greater after 1 hour in both groups, with rates if 64.3% and 64.6% in the fluoroscopy and anatomical parameters groups respectively, with no significant difference. At the end of 90 days, the pain reduction rates were of 35.7% and 44.6% in the fluoroscopy and anatomical parameter groups respectively. The VAS also showed a significant reduction ( *p* < 0.05) in pain in both groups at all times. The reduction was more significant after 1 hour: 78.0% in the fluoroscopy group and 82.2% in the anatomical parameter group, with no significant difference. At the end of 90 days, the pain reduction was of in the fluoroscopy group 36.5% and of 24.6% in the anatomical parameters group.

**Conclusion** The GNBs guided by fluoroscopy or by anatomical parameters alone were equally effective in terms of magnitude and duration of pain relief.

## Introduction

Knee osteoarthritis (KOA) is the most prevalent chronic joint disease, 1 2 affecting 12% of subjects over 60. 3 Its prevalence rate increases with age, ranging from 4.2 to 15.5. Approximately 80% of KOA patients are aged 65 years or older. Diagnosis may rely on imaging methods, such as weight-bearing plain radiographs.

Around 60% of patients with KOA present clinical manifestations, 4 5 especially joint stiffness, and muscle atrophy. There is a 25% rate of patients presenting with severe, limiting arthralgia. 6 Treatment includes noninvasive clinical therapy with medications, physical therapy, and rehabilitation, and minimally invasive treatment with intra- and periarticular injections. 7 Many patients suffer from chronic, incapacitating pain refractory to clinical treatment. For these cases, surgical treatment is indicated, including arthroscopy, osteotomies, and arthroplasties with implants, depending on patients' age and the degree of joint involvement. 8

As an alternative to surgical treatment, Choi et al. 9 described the genicular nerve block (GNB) technique, which has been effective in relieving pain and improving joint functionality in KOA patients. 9 10 11 12 13 Therefore, it is a new option for relieving refractory chronic pain in these subjects. There are four genicular nerves: superomedial, superolateral, inferomedial, and inferolateral. The traditional technique blocks all nerves, except for the inferolateral genicular nerve due to its proximity to the common fibular nerve and risk of neuropraxia. 14

Ultrasound and fluoroscopy are two imaging methods that can increase the accuracy of the technique, aiding in determining anatomical landmarks. Cadaveric studies clarified the location of the genicular nerves (origin, termination, course) and their anatomical relationship with the surrounding tissues. 15 16 17 Interestingly, this detailed description prompted several studies evaluating GNB performance using imaging. 10 12 18 Additionally, it is possible to perform the procedure using anatomical parameters alone, with no need for auxiliary imaging methods. 14

To date, ultrasound-guided procedures have not proven consistently superior to their fluoroscopy counterparts, 19 which is also more accessible, easier for the orthopedist, and does not require additional expertise in musculoskeletal ultrasound. As such, the main objective of this study was to compare the effectiveness of fluoroscopy versus anatomical parameters (with no radiological resources) as an auxiliary method in GNB for pain relief in KOA patients.

## Materials and methods

The institutional Ethics Committee approved the study under number (58941322.5.0000.5505).

The present study was a quasi-randomized clinical trial and patient selection for blocks with and without image assistance occurred on a first-come, first-served basis. We collected demographic data from participants, including age at the time of the procedure, gender, side of the lesion, and body mass index (BMI).

All data were obtained directly from the patient or the electronic medical record after the researchers applied the informed consent form.

### Selection criteria

#### Inclusion criteria

The study included patients from 50 to 80-years-old; osteoarthritis with pain for at least 3 months; having knee radiographs demonstrating Kellgren-Lawrence 3 or 4 osteoarthritis; and/or symptomatic osteoarthritis refractory to clinical treatment involving oral medications (such as analgesics, nonsteroidal anti-inflammatory drugs [NSAIDs] etc.), and physical therapy for at least 6 months.

#### Exclusion criteria

The study excluded patients with: severe neurological or psychiatric diseases; history of steroid injections within the last 3 months; sciatica; previous knee surgeries, including knee replacement (total or unicompartmental), debridement arthroscopy, or osteotomies; and those who make use of anticoagulants. We also excluded patients who did not receive the scheduled intervention; lost to follow-up before 3 months; and those with any adverse reaction to the procedure. In this last case, however, despite being excluded from the final analysis, we documented and reported any adverse reactions observed during the study.

### Genicular nerve block

After using the reference methods according to the predefined group (fluoroscopy or anatomical parameters), each patient underwent the GNB procedure with a solution containing 8 mL of methylprednisolone (500 mg), 6 mL of 0.5% bupivacaine, 1 mL of clonidine (150 μg), and a saline solution to a final volume of 20 mL, with 5 mL applied at each point.

#### Fluoroscopy-guided

**Fig. 1:**
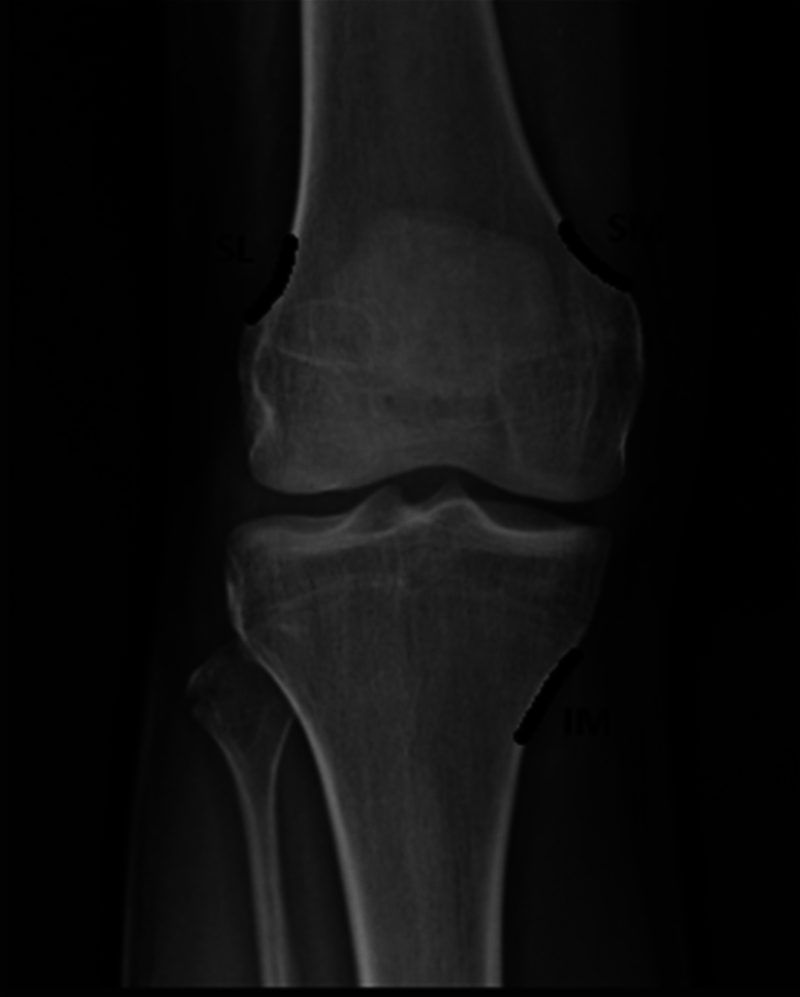
Anteroposterior radiograph demonstrating the reference points at fluoroscopy. **Abbreviations:** SL, superolateral; SM, superomedial; IM, inferomedial.

Through the anteroposterior view, we identified three reference points: the medial and lateral transition areas of the femoral cortex, with the respective femoral condyles and the transition area between the medial tibial condyle and tibial diaphysis ( Fig. 1 ). 19

#### Anatomical parameter-based

**Fig. 2:**
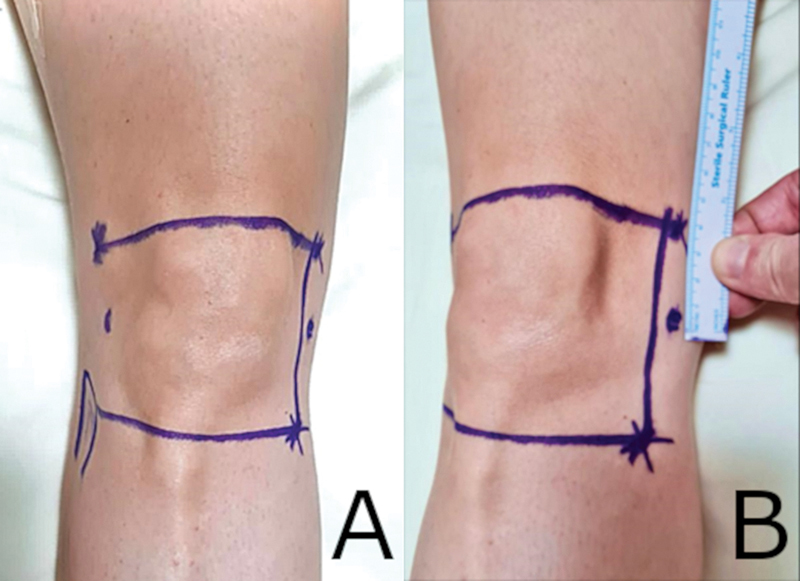
( **A** ) Anterior view with lines drawn through the head of the fibula; ( **B** ) oblique view showing the line drawn 4 cm above the femoral epicondyle.

We drew a longitudinal line from the fibular head towards the femur, extending up to 4 cm superior to the lateral femoral epicondyle. Then, another line was drawn horizontally between the femoral epicondyles, and a third one from the medial femoral epicondyle to the medial tibial epicondyle. These were the reference points. We applied the solution at the line intersections, 14 as shown in Fig. 2 .

### Outcome measures

We administered the Visual Analog Scale (VAS) for pain and the pain subscale of the Western Ontario and McMaster Universities Osteoarthritis Index (WOMAC) to patients enrolled in the study before undergoing GNB and at the following time points: 1 hour, 24 hours, 7 days, 28 days, and 90 days after the procedure. The questions were read to the patients in person or by telephone, if they could not return for follow-up appointments in person. Both questionnaires were translated into Portuguese and validated for the Brazilian context. 20 21

The outcome assessment was single blinded since the professional who performed the GNB was not the same one evaluating the data. Additionally, the professional in charge of the assessment was unaware of which group the patients belonged to.

### Statistical analysis

After data collection, the analysis used the IBM SPSS Statistics for Windows (IBM Corp., Armonk, NY, United States) software, version 23.0. Descriptive statistics determined the clinical and sociodemographic characteristics of patients. The Shapiro-Wilk test assessed continuous variables. Analysis and comparisons between preoperative and postoperative clinical-functional scores used the Wilcoxon test (nonparametric; non-normally-distributed variables) and the paired *t* test (parametric; normally-distributed variables) per sampling distribution. Comparisons of self-administered clinical-functional scores in the primary analysis with the secondary analysis and other continuous variables (age, follow-up, BMI) employed the unpaired *t* test (parametric; normally-distributed variables) or the Mann-Whitney *U* test (nonparametric; non-normally-distributed variables) based on the sampling distribution. The Chi-squared test evaluated the distribution of categorical variables between the groups.

## Results

Initially, 27 patients met the inclusion criteria and underwent the GNB guided by radioscopy or clinical parameters. There were 4 patients (14.8%) lost to follow-up before completing 3 months, which were excluded from the study. Therefore, the final sample consisted of 23 patients, with a total follow-up of 85.2%, and a mean age of 64.5 ± 4.8 years. There were 16 (69.6%) females and the mean BMI was of 31.4 ± 6.1 Kg/m ^2^ . We divided the eligible patients into two groups: the first group had 12 (52.2%) patients who underwent fluoroscopy-guided GNB; and the second group had 11 (47.8%) patients underwent a procedure based on anatomical parameters, that is, with no radiological aid. There were no statistically significant differences in the distribution of demographic variables between the groups ( Table 1 ).

**Table 1:** Demographic characteristics of the study sample

Characteristics	Total (N = 23)	Fluoroscopy (N = 12)	Anatomical Parameter (N = 11)	*p-* value
Age (years): mean ± SD	64.5 ± 4.8	65.3 ± 4.4	63.9 ± 5.2	0.539
Gender: n (%)				
Male	7 (30.4)	4 (36.4)	3 (25.0)	0.667
Female	16 (69.6)	7 (63.6)	9 (75.0)	0.444
BMI (Kg/m ^2^ ): mean ± SD	31.4 ± 6.1	29.4 ± 6.2	33.1 ± 5.8	0.189

**Abbreviations:** BMI, Body Mass Index; GNB, genicular nerve block; SD, standard deviation.

**Fig. 3:**
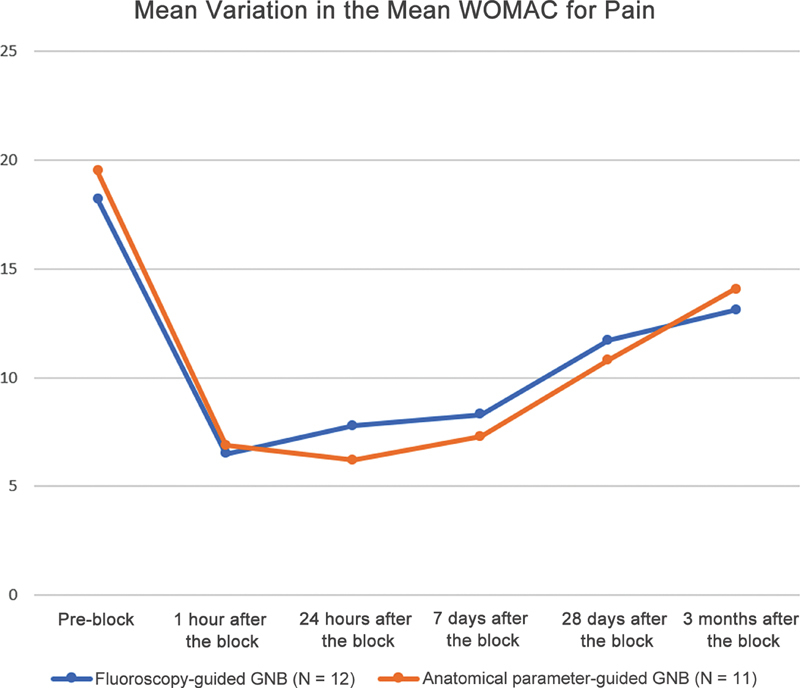
Graphical representation of the variation in the mean score on the pain subscale of the Western Ontario and McMaster Universities Osteoarthritis Index (WOMAC) at the different evaluation times. **Note:** The mean pos-block scores are statistically lower than the mean preblock score for all evaluation times ( *p* < 0.05). **Abbreviation:** GNB, genicular nerve block.

After analyzing the WOMAC pain subscale questionnaires, we observed a statistically significant improvement ( *p* < 0.05) at all times after GNB compared with the preprocedure pain level. The most representative improvement was closer to the procedure. We detected no significant differences between the outcomes from the two GNB types at any time point studied ( Table 2 ). In the fluoroscopy group, the preprocedure pain level was 18.2 ± 3.9 of the 20 potential points. In the anatomical parameters group, it was 19.5 ± 1.3. We noted the highest reduction in pain 1 hour after the procedure, with an improvement of 11.7 ± 3.8 (64.3%, *p* = 0.003) points in the fluoroscopy group, and in the anatomical parameters group, of 13.3 ± 1.9 (68.2%, *p* = 0.002) after 24 hours. During the follow-up, we observed a tendency for increased pain. After 90 days of the procedure, the reduction in pain in the fluoroscopy group was 5.1 ± 6.0 ( *p* = 0.024) points (35.7%) and, in the anatomical parameters group, it was 5.3 ± 3.2 ( *p* < 0.001) points (44.6%) ( Fig. 3 ).

**Table 2:** Comparison of the mean score of the study sample on the pain subscale of the WOMAC at different assessment times

Assessment time	Group		
	Fluoroscopy (N = 12)	Anatomical Parameter (N = 11)	*p-* value
Pre-GNB (mean ± SD)	18.2 ± 3.9	19.5 ± 1.3	0.478
Post-GNB (mean ± SD)/Improvement ( *p-* value for improvement)			
1 hour	6.5 ± 2.1/11.7 ± 3.8 ( *p =* 0.003)	6.9 ± 2.4/12.5 ± 1.9 ( *p =* 0.002)	0.566
24 hours	7.8 ± 3.4/10.4 ± 5.0 *(p =* 0.003)	6.2 ± 1.8/13.3 ± 1.9 ( *p =* 0.002)	0.347
7 days	8.3 ± 3.4/9.9 ± 5.5 ( *p* = 0.007)	7.3 ± 1.9/12.1 ± 2.2 ( *p* = 0.003)	0.654
28 days	11.7 ± 2.8/6.5 ± 3.8 ( *p* < 0.001)	10.8 ± 3.9/8.6 ± 4.0 ( *p* < 0.001)	0.423
3 months	13.1 ± 3.6/5.1 ± 6.0 ( *p* = 0.024)	14.1 ± 3.2/5.3 ± 3.2 ( *p* < 0.001)	0.401

**Abbreviations:** GNB, genicular nerve block; SD, standard deviation; WOMAC, Western Ontario and McMaster Universities Osteoarthritis Index.

**Note:** “Improvement” refers to the comparison with the mean score at the preblock assessment time.

**Fig. 4:**
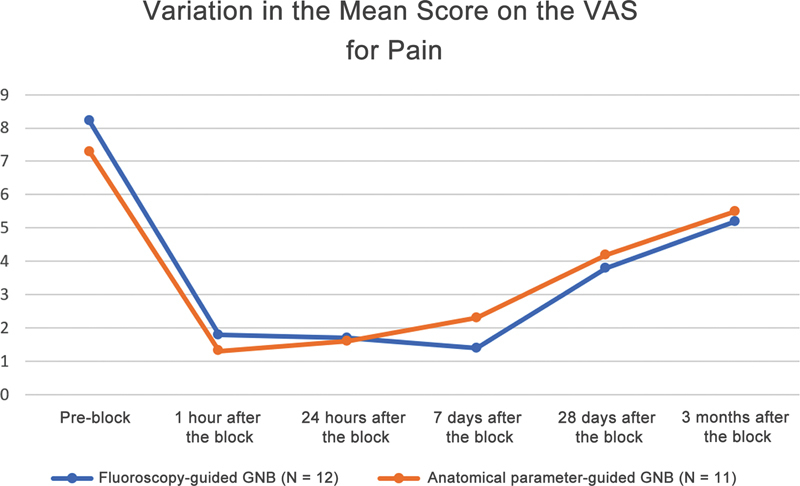
Graphical representation of the variation in the mean score on the visual analog scale (VAS) for pain at the different evaluation times. **Note:** The mean postblock scores are statistically lower when compared to the mean preblock score for all assessment times ( *p* < 0.05). **Abbreviation:** GNB, genicular nerve block.

The VAS analysis revealed a statistically significant improvement ( *p* < 0.05) at all times after the procedure when compared with the pre-procedure pain level. The more expressive improvement was closer to the procedure ( Table 3 ). There was no significant difference between the groups at any time point studied. The pain reduction after 1 hour of the procedure was more intense, with a score of 6.5 ± 1.4 (78.0%, *p* = 0.003) of the 10 potential points in the fluoroscopy group and 6.1 ± 2.7 (82.2%, *p* = 0.002) in the anatomical parameters group. We noted a trend for increased pain throughout the postprocedure follow-up. Despite this trend, at the end of the 90-day follow-up, there was a pain reduction of 3.1 ± 1.6 (36.5%, *p* = 0.005) points in the fluoroscopy group and 1.8 ± 2.4 (24.6%, *p* = 0.032) in the anatomical parameters group ( Fig. 4 ).

**Table 3:** Comparison of the mean scores of the study sample on the VAS for pain at different assessment times

Assessment time	Group		
	Fluoroscopy (N = 12)	Anatomical Parameter (N = 11)	*p-* value
Pre-GNB (mean ± SD)	8.2 ± 0.8	7.3 ± 2.3	0.566
Post-GNB (mean ± SD)/Improvement ( *p-* value for improvement)			
1 hour	1.8 ± 1.3/6.5 ± 1.4 ( *p* = 0.003)	1.3 ± 1.8/6.1 ± 2.7 ( *p* = 0.002)	0.235
24 hours	1.7 ± 2.4/6.5 ± 2.5 ( *p* = 0.005)	1.6 ± 1.7/5.8 ± 3.1 ( *p* = 0.003)	0.999
7 days	1.4 ± 1.6/6.9 ± 1.9 ( *p* = 0.003)	2.3 ± 1.5/5.0 ± 2.7 ( *p* = 0.003)	0.134
28 days	3.8 ± 1.3/4.5 ± 0.9 ( *p* = 0.002)	4.2 ± 1.6/3.2 ± 2.7 ( *p* = 0.007)	0.563
3 months	5.2 ± 1.4/3.1 ± 1.6 ( *p* = 0.005)	5.5 ± 1.2/1.8 ± 2.4 ( *p* = 0.032)	0.449

**Abbreviations:** GNB, genicular nerve block; SD, standard deviation; VAS, Visual Analog Scale.

**Note:** “Improvement” refers to the comparison with the mean score at the preblock assessment time.

## Discussion

Cankurtaran et al. 14 compared the efficacy of blockades guided by ultrasound and anatomical parameters, showing efficacy in pain reduction and function improvement, with similar outcomes in both techniques. Kim et al. 19 compared the efficacy of ultrasound versus fluoroscopy guided blockades, and pain relief, functional improvement, and safety were consistent between the groups. However, considering radiation exposure, ultrasound may be superior to fluoroscopic guidance. The results of this study confirmed the efficacy of GNB in reducing pain in patients with KOA, regardless of the method used. These findings are consistent with other studies 14 19 from different countries and ethnic profiles, suggesting the external validity of the findings. Pain reduction was more significant immediately after the procedure and gradually decreased over 90 days.

This study also demonstrated the non-superiority of fluoroscopy-guided GNB compared with anatomic parameter-based GNB with no radiological resources. This is particularly relevant in centers with relatively limited budgets and infrastructure, showing that radiological methods are not required for an effective procedure. Another factor to consider is radiation exposure, which is not present in anatomic parameter-based GNB.

These data were consistent even when different surgeons with distinct experience levels performed the GNB, which indicates that this technique has a short learning curve and predictable outcomes.

We observed a trend toward increased pain levels over the 90-day follow-up period, although maintaining a significant reduction compared with preprocedure levels. These findings agree with those of the international literature 14 18 19 and suggest that, at a certain point, a new GNB would be required for symptom control.

Study limitations included not achieving the minimum sample of 56 patients, calculated in the preproject. Even so, we could achieve statistical significance in all postprocedure periods. Additionally, we did not perform multivariate analysis for each comorbidity, their relationships with preprocedure pain, and impacts on the respective outcomes as independent variables. Due to the scarcity of studies on this topic, future investigations are necessary to better correlate the outcomes and risk factors.

## Conclusion

We observed no statistically significant differences ( *p* < 0.05) between fluoroscopy-guided GNB and anatomic parameter-based GNB regarding pain relief in patients with KOA.

Therefore, the choice of technique is at the physicians or patients' discretion for GNB in KOA.
