# Supplementary material for: Efficacy of Genicular Nerve Block in Patients with Osteoarthritis: A Comparative Study with and without Fluoroscopy Assistance
Source: Rev Bras Ortop (Sao Paulo). 2024 Dec 21;59(6):e876–82. doi: 10.1055/s-0044-1793825 (PMC11663072; doi:10.1055/s-0044-1793825)
Supplement: Related texts

# Research article: Eficácia do bloqueio dos nervos geniculares em pacientes com osteoartrite: Um estudo comparativo com e sem auxílio de fluoroscopia

**Keywords:** bloqueio nervoso, dor, escala visual analógica da dor, fluoroscopia, osteoartrite de joelho

## Abstract

**Objetivo** Comparar a eficácia da fluoroscopia como método auxiliar no bloqueio dos nervos geniculares (BNG) com o bloqueio guiado por parâmetros anatômicos, sem auxílio de imagem na redução da dor.

**Métodos** No total, 23 pacientes foram submetidos ao BNG com o auxílio de fluoroscopia ou de parâmetros anatômicos. O Western Ontario and McMaster Universities Osteoarthritis Index (WOMAC) e a Escala Visual Analógica (EVA) foram aplicados em 6 momentos (pré-bloqueio, após 1 hora, 24 horas, 7 dias, 28 dias e 90 dias).

**Resultados** A média de idade da amostra foi de 64,5 ± 4,8 anos, e o Índice de Massa Corporal (IMC), de 31,4 ± 6,1 Kg/m ^2^ ; 16 pacientes (69,6%) eram mulheres. Pela subescala da dor do WOMAC, foi obtida redução significativa ( *p* < 0,05) da dor nos dois grupos em todos os momentos. Tal redução foi maior após 1 hora em ambos os grupos, com taxas de 64,3% e 64,6% no grupo de fluoroscopia e de parâmetros anatômicos, respectivamente, sem diferença significativa. Ao final de 90 dias, foi obtida uma redução da dor de 35,7% e 44,6% nos grupos de fluoroscopia e de parâmetros anatômicos, respectivamente. Pela EVA, também foi observada redução significativa ( *p* < 0,05) da dor em ambos os grupos e em todos os momentos. A redução foi mais importante após 1 hora: 78,0% no grupo de fluoroscopia e 82,2% no grupo de parâmetros anatômicos, sem diferença significativa. Ao final de 90 dias, foi obtida uma redução da dor de 36,5% no grupo de fluoroscopia e de 24,6% no grupo de parâmetros anatômicos respectivamente.

**Conclusão** Os BNGs guiados por fluoroscopia ou apenas por parâmetros anatômicos foram igualmente eficazes em termos de magnitude ou duração de alívio da dor.

## Introdução

A osteoartrite de joelho (OAJ) é a doença articular crônica de maior prevalência, 1 2 afetando 12% dos indivíduos com mais de 60 anos. 3 Sua taxa de prevalência aumenta com a idade variando entre 4,2 e 15,5. Aproximadamente 80% dos portadores de OAJ possuem 65 anos ou mais. O diagnóstico pode ser feito por meio de métodos de imagem, como radiografias simples com carga, por exemplo.

Cerca de 60% dos pacientes com OAJ apresentam manifestação clínica da doença, 4 5 principalmente dor, rigidez articular e atrofia muscular. Um total de 25% dos pacientes apresenta artralgia grave limitante. 6 O tratamento inclui o tratamento clínico não invasivo com medicamentos, fisioterapia e reabilitação; e o minimamente invasivo com infiltrações intra e periarticulares. 7 Diversos pacientes sofrem de dor crônica incapacitante refratária ao tratamento clínico. Nesses casos o tratamento cirúrgico passa a ser indicado, incluindo artroscopia, osteotomias e as artroplastias com implante, a depender da idade do paciente e do grau de acometimento articular. 8

Como alternativa ao tratamento cirúrgico, Choi et al. 9 descreveram a técnica do bloqueio dos nervos geniculares (BNG). Esta tem se mostrado efetiva no alívio da dor, melhorando a funcionalidade da articulação dos pacientes com OAJ. 9 10 11 12 13 Portanto, tem se tornado uma nova opção no alívio das dores crônicas refratárias nesses pacientes. Existem quatro nervos geniculares: superomedial, superolateral, inferomedial e inferolateral. Na técnica tradicional todos são bloqueados, com exceção do n. genicular inferolateral devido sua proximidade ao nervo fibular comum e risco de neuropraxia. 14

Ultrassom e fluoroscopia são dois métodos de imagem que podem ser utilizados para aumentar a precisão da técnica, auxiliando na determinação dos pontos de referência anatômicos. Após estudo em cadáveres, foi esclarecido a localização dos nervos geniculares (origem, terminação, trajeto) e o seu relacionamento anatômico com os tecidos circundantes. 15 16 17 Curiosamente, essa descrição detalhada impulsionou diversos estudos avaliando o desempenho do BNG com uso de recursos de imagem. 10 12 18 Também é possível realizar o procedimento utilizando apenas parâmetros anatômicos, sem a necessidade de métodos de imagem auxiliares. 14

Até o momento, o procedimento guiado por ultrassom não se provou consistentemente superior a fluoroscopia, 19 que também é mais acessível, mais familiar ao ortopedista e não requer expertise adicional em ultrassonografia musculoesquelética. Frente a tal panorama, o objetivo principal deste estudo é comparar a eficácia da fluoroscopia versus parâmetros anatômicos (sem recurso radiológico) como método auxiliar no BNG no alívio da dor em pacientes com OAJ.

## Materiais e Métodos

O estudo foi aprovado pelo Comitê de Ética institucional sob o número (58941322.5.0000.5505).

O presente estudo foi conduzido como um ensaio clínico quase-randomizado, no qual a seleção dos pacientes para os bloqueios com e sem auxílio de imagem foi realizado por livre demanda, seguindo a ordem de chegada. Os dados demográficos coletados dos participantes foram idade no momento do procedimento, sexo, lateralidade da lesão e Índice de Massa Corporal (IMC).

Todos os dados demográficos foram informados diretamente pelo paciente ou coletados do prontuário eletrônico após aplicação do termo de consentimento livre e esclarecido (TCLE) pelos pesquisadores.

## Critérios de seleção

## Critérios de inclusão

Foram incluídos no estudo pacientes com idade de 50 a 80 anos; osteoartrite com ao menos 3 meses de dor; radiografias de joelhos demonstrando osteoartrite de graus 3 ou 4 na classificação de Kellgren-Lawrence; osteoartrite sintomática refratária ao tratamento clínico com medicações de via oral (como analgésicos, anti-inflamatórios não esteroides [AINES] etc.) e fisioterapia por pelo menos 6 meses.

## Critérios de exclusão

Foram excluídos do estudo os pacientes que apresentavam: doenças neurológicas graves ou psiquiátricas; histórico de infiltração com esteroides nos últimos 3 meses; ciatalgia; cirurgia prévia no joelho, incluindo prótese de joelho (total ou unicompartimental), artroscopia toalete ou osteotomias; e aqueles que fizeram uso de anticoagulante. Também foram excluídos os pacientes que: não receberam a intervenção programada; perderam o seguimento antes de completar 3 meses; e apresentaram qualquer tipo de reação adversa ao procedimento realizado. Nesse último caso, entretanto, mesmo excluídos da análise final, toda e qualquer reação adversa observada durante a realização do estudo foi documentada e relatada nos resultados.

## Bloqueio dos nervos geniculares

Após o uso dos métodos de referência de acordo com o grupo predefinido (fluoroscopia ou parâmetros anatômicos), cada paciente foi submetido ao procedimento de bloqueio nervoso genicular, através de uma solução de: 1 frasco de metilprednisolona (500 mg) contendo 8 mL; 6 mL de bupivacaína a 0,5%; 1 frasco de clonidina (150 μg) contendo 1 mL; e completa com 20 mL de solução com soro fisiológico, sendo aplicado 5 mL em cada ponto.

## Com auxílio de fluoroscopia

Por meio da incidência anteroposterior, três pontos de referência foram buscados: áreas medial e lateral de transição da cortical femoral, com os respectivos côndilos femorais, e área de transição entre côndilo tibial medial e diáfise tibial ( Fig. 1 ). 19

**Fig. 1:** Radiografia anteroposterior demonstrando os pontos de referência a serem buscados com auxílio da fluoroscopia. **Abreviaturas:** SL, superolateral; SM, superomedial; IM, inferomedial.

## Com parâmetros anatômicos

Foi traçada uma linha longitudinalmente através da cabeça fibular em direção ao fêmur, que se estende até 4 cm superior ao epicôndilo femoral lateral; outra linha traçada horizontalmente entre os epicôndilos femorais e uma terceira linha foi traçada do epicôndilo femoral medial até o epicôndilo tibial medial, sendo estes os pontos de referência. Com as linhas tracejadas, a aplicação da solução foi realizada nas intersecções das linhas 14 ( Fig. 2 ).

**Fig. 2:** ( **A** ) Visão anterior com as linhas traçadas através da cabeça da fíbula; ( **B** ) visão obliqua evidenciando a linha traçada 4 cm acima do epicôndilo femoral.

## Medidas de desfecho

A Escala Visual Analógica (EVA) para dor e a subescala de dor do Western Ontario and McMaster Universities Osteoarthritis Index (WOMAC) foram aplicadas aos pacientes envolvidos no estudo antes da realização do BNG e nos seguintes momentos: 1 hora, 24 horas, 7 dias, 28 dias e 90 dias após o procedimento. Os questionários foram aplicados por meio da leitura das perguntas aos pacientes de forma presencial ou por meio do telefone nos casos em que não puderam comparecer pessoalmente às consultas de retorno. Ambos os questionários foram traduzidos e validados para o português e realidade brasileira. 20 21

A avaliação dos resultados foi conduzida de forma simples cega, uma vez que o profissional responsável pela execução do bloqueio não foi o mesmo que realizou a avaliação dos dados. Além disso, o profissional encarregado das avaliações não tinha conhecimento a respeito de a qual grupo pertenciam os pacientes.

## Análise estatística

Após a coleta dos dados, a análise foi realizada com o programa IBM SPSS Statistics for Windows (IBM Corp., Armonk, NY, Estados Unidos), versão 23.0. Características clínicas e sociodemográficas dos pacientes foram determinadas por estatística descritiva. O teste de Shapiro-Wilk foi utilizado nas variáveis contínuas. A análise e comparações entre os escores clínico-funcionais pré- e pós-operatórios foram avaliados por meio dos testes de Wilcoxon (não paramétrico; variáveis de distribuição não normal) e *t* pareado (paramétrico; variáveis de distribuição normal), de acordo com a distribuição amostral. As comparações dos escores clínico-funcionais autoaplicados nas análises primária, com a análise secundária e outras variáveis contínuas (idade, seguimento, IMC) foram realizadas utilizando-se os testes *t* não pareado (paramétrico; variáveis de distribuição normal) ou *U* de Mann-Whitney (não paramétrico; variáveis de distribuição não normal), com base na distribuição amostral. O teste do Qui-quadrado foi utilizado para avaliar a distribuição de variáveis categóricas entre os grupos.

## Resultados

Inicialmente, 27 pacientes satisfizeram os critérios de inclusão e foram submetidos ao BNG auxiliado por radioscopia ou parâmetros clínicos. Foram 4 pacientes (14,8%) apresentando perda de seguimento antes de completar 3 meses, precisando ser excluídos. Portanto, 23 pacientes compuseram a amostra final, totalizando o seguimento total de 85,2%, com a média de idade de 64,5 ± 4,8 anos. Do total, 16 (69,6%) eram mulheres e o IMC médio foi 31,4 ± 6,1 Kg/m ^2^ . Os pacientes elegíveis foram separados em dois grupos: o primeiro grupo, com 12 (52,2%) pacientes, foi submetido ao BNG com auxílio da fluoroscopia; e o segundo grupo, com 11 (47,8%) pacientes, foi submetido ao procedimento com auxílio de parâmetros anatômicos, sem método radiológico de auxílio. Não houve diferenças estatisticamente significantes na distribuição das variáveis demográficas entre os grupos ( Tabela 1 ).

**Tabela 1:** Características demográficas da amostra do estudo

Características	Total (N = 23)	Fluoroscopia (N = 12)	Parâmetros anatômicos (N = 11)	Valor de *p*
Idade (anos): média ± DP	64,5 ± 4,8	65,3 ± 4,4	63,9 ± 5,2	0,539
Sexo: n (%)				
Masculino	7 (30,4)	4 (36,4)	3 (25,0)	0,667
Feminino	16 (69,6)	7 (63,6)	9 (75,0)	0,444
IMC (Kg/m ^2^ ): média ± DP	31,4 ± 6,1	29,4 ± 6,2	33,1 ± 5,8	0,189

**Abreviaturas:** BNG, bloqueio dos nervos geniculares; DP, desvio padrão; IMC, Índice de Massa Corporal.

Após análise dos questionários da subescala da dor do WOMAC, observou-se uma melhora estatisticamente significativa ( *p* < 0,05) em todos os momentos após o procedimento, quando se compara com o nível da dor pré-procedimento; com melhora mais representativa quanto mais próximo do procedimento. Não foi detectada diferença significativa entre os resultados nos dois tipos de BNG em nenhum momento estudado ( Tabela 2 ). No grupo fluoroscopia o nível da dor pré-procedimento era de 18,2 ± 3,9 dos 20 pontos possíveis. No grupo parâmetros anatômicos era de 19,5 ± 1,3. A maior redução da dor foi obtida 1 hora pós-procedimento, com melhora de 11,7 ± 3,8 (64,3%, *p =* 0,003) pontos no grupo fluoroscopia e após 24 horas no grupo parâmetros anatômicos 13,3 ± 1,9 (68,2%, *p =* 0,002) pontos. Ao longo do acompanhamento foi observada tendência de aumento da dor. Após 90 dias do respectivo procedimento, foi obtido uma redução na dor de 5,1 ± 6,0 (35,7%, *p* = 0,024) pontos no grupo fluoroscopia e de 5,3 ± 3,2 (44,6%, *p* < 0,001) pontos no grupo parâmetros anatômicos ( Fig. 3 ).

**Fig. 3:** Representação gráfica da variação da média da pontuação na subescala de dor do Western Ontario and McMaster Universities Osteoarthritis Index (WOMAC) nos diferentes momentos de avaliação. **Nota:** As médias das pontuações pós-bloqueio são estatisticamente menores quando comparadas com a média da pontuação pré-bloqueio para todos os momentos de avaliação ( *p* < 0,05).

**Tabela 2:** Comparação das pontuações médias da amostra do estudo na subescala de dor do WOMAC nos diferentes momentos de avaliação

Momento de avaliação	Grupo		Valor de *p*
	Fluoroscopia (N = 12)	Parâmetros anatômicos (N = 11)	
Pré-bloqueio (média ± DP)	18,2 ± 3,9	19,5 ± 1,3	0,478
Pós-bloqueio (média ± DP)/Melhora (valor de *p* da melhora)			
1 hora	6,5 ± 2,1/11,7 ± 3,8 ( *p =* 0,003)	6,9 ± 2,4/12,5 ± 1,9 ( *p =* 0,002)	0,566
24 horas	7,8 ± 3,4/10,4 ± 5,0 *(p =* 0,003)	6,2 ± 1,8/13,3 ± 1,9 ( *p =* 0,002)	0,347
7 dias	8,3 ± 3,4/9,9 ± 5,5 ( *p* = 0,007)	7,3 ± 1,9/12,1 ± 2,2 ( *p* = 0,003)	0,654
28 dias	11,7 ± 2,8/6,5 ± 3,8 ( *p* < 0,001)	10,8 ± 3,9/8,6 ± 4,0 ( *p* < 0,001)	0,423
3 meses	13,1 ± 3,6/5,1 ± 6,0 ( *p* = 0,024)	14,1 ± 3,2/5,3 ± 3,2 ( *p* < 0,001)	0,401

**Abreviaturas:** DP, desvio padrão; WOMAC, Western Ontario and McMaster Universities Osteoarthritis Index.

**Nota:** “Melhora” refere-se à comparação com a média da pontuação no momento de avaliação pré-bloqueio.

Seguindo a análise do questionário EVA, houve melhora estatisticamente significativa ( *p* < 0,05) em todos os momentos após o procedimento, quando se compara ao nível da dor pré-procedimento; com melhora mais expressiva quanto mais próximo do procedimento ( Tabela 3 ). Não houve diferença significativa entre os grupos em nenhum momento estudado. A redução da dor após 1 hora dos respectivos procedimentos foi mais intensa, com redução de 6,5 ± 1,4 (78,0%, *p* = 0,003) pontos dos 10 possíveis no grupo fluoroscopia, e de 6,1 ± 2,7 (82,2%, *p* = 0,002) pontos da dor no grupo dos parâmetros anatômicos. A tendência era de aumento da dor ao longo do acompanhamento pós-procedimento. Apesar dessa tendência, ao final do acompanhamento de 90 dias, houve redução da dor de 3,1 ± 1,6 (36,5%, *p* = 0,005) pontos no grupo fluoroscopia e de 1,8 ± 2,4 (24,6%, *p* = 0,032) no grupo parâmetros anatômicos ( Fig. 4 ).

**Fig. 4:** Representação gráfica da variação da pontuação média na escala visual analógica de dor nos diferentes momentos de avaliação. **Nota:** As médias das pontuações pós-bloqueio são estatisticamente menores quando comparadas com a média da pontuação pré-bloqueio para todos os momentos de avaliação ( *p* < 0,05).

**Tabela 3:** Comparação das pontuações médias da amostra do estudo na EVA para dor nos diferentes momentos de avaliação

Momento de avaliação	Grupo		Valor de *p*
	Fluoroscopia (N = 12)	Parâmetros anatômicos (N = 11)	
Pré-bloqueio (média ± DP)	8,2 ± 0,8	7,3 ± 2,3	0,566
Pós-bloqueio (média ± DP)/Melhora (valor de *p* da melhora)			
1 hora	1,8 ± 1,3/6,5 ± 1,4 ( *p* = 0,003)	1,3 ± 1,8/6,1 ± 2,7 ( *p* = 0,002)	0,235
24 horas	1,7 ± 2,4/6,5 ± 2,5 ( *p* = 0,005)	1,6 ± 1,7/5,8 ± 3,1 ( *p* = 0,003)	0,999
7 dias	1,4 ± 1,6/6,9 ± 1,9 ( *p* = 0,003)	2,3 ± 1,5/5,0 ± 2,7 ( *p* = 0,003)	0,134
28 dias	3,8 ± 1,3/4,5 ± 0,9( *p* = 0,002)	4,2 ± 1,6/3,2 ± 2,7 ( *p* = 0,007)	0,563
3 meses	5,2 ± 1,4/3,1 ± 1,6 ( *p* = 0,005)	5,5 ± 1,2/1,8 ± 2,4 ( *p* = 0,032)	0,449

**Abreviaturas:** DP, desvio padrão; EVA, Escala Visual Analógica.

**Nota:** “Melhora” refere-se à comparação com a pontuação média no momento de avaliação pré-bloqueio.

## Discussão

Cankurtaran et al. 14 compararam a eficácia do bloqueio guiado por ultrassom e por parâmetros anatômicos, apresentando eficácia na redução da dor e melhora funcional, com resultados semelhantes em ambas as técnicas. Kim et al. 19 compararam a eficácia do bloqueio guiado por ultrassom versus fluoroscopia, onde o alívio da dor, a melhora funcional e a segurança foram semelhantes entre os grupos. No entanto, considerando exposição à radiação, a orientação ultrassonográfica pode ser superior à orientação fluoroscópica. Os resultados deste estudo confirmaram a eficácia do BNG na redução da dor em pacientes com osteoartrite do joelho, independentemente do método utilizado. Esses achados estão em consonância com outros estudos 14 19 realizados em diferentes países e com diferentes padrões étnicos, sugerindo validade externa dos achados. A redução da dor for mais significativa logo após o procedimento e diminuiu gradualmente ao longo dos 90 dias.

Neste estudo, também foi demonstrada a não superioridade do BNG com o auxílio de fluoroscopia ao BGN realizado por parâmetros anatômicos, sem recursos radiológicos. Tendo grande relevância especialmente em centros com recursos orçamentários e infraestrutura relativamente limitados, mostrando que não é necessário dispor de métodos radiológicos para a realização do procedimento com eficácia. Outro fator a se considerar é a exposição à radiação, que é inexistente no BNG por parâmetros anatômicos.

Tais dados se mostraram consistentes mesmo com a realização dos BNG por diferentes cirurgiões de diferentes níveis de experiência, indicando se tratar de uma técnica com curva de aprendizagem curta e de resultados previsíveis.

Também foi observada uma tendência de aumento dos patamares álgicos ao longo dos 90 dias de seguimento, mantendo, contudo, a redução significativa quando comparado aos patamares pré-procedimento. Esses achados estão de acordo com a literatura internacional, 14 18 19 sugerindo que, em um certo ponto, seria necessário realizar novamente o procedimento para controle dos sintomas.

Dentre as limitações do estudo, não foi possível atingir a amostra mínima de 56 pacientes, calculado no pré-projeto como objetivo. Ainda assim, foi possível atingir significância estatística em todos os períodos pós-procedimento. Adicionalmente, não foi realizada análise multivariada de cada comorbidade, suas relações com a dor pré-procedimento e seus impactos nos respectivos resultados como variáveis independentes. Devido à escassez de trabalhos nesse sentido, torna-se necessário estudos futuros para melhor correlação dos resultados e fatores de risco.

## Conclusão

Não foram observadas diferenças estatisticamente significativas ( *p* < 0,05) entre o BNG realizado com fluoroscopia versus com parâmetros anatômicos em termos de alívio da dor em pacientes com OAJ.

Portanto, a escolha de técnica pode ficar a critério do médico-paciente para BNG em pacientes com OAJ.
